# Prostate Cancer Incidence under Androgen Deprivation: Nationwide Cohort Study in Trans Women Receiving Hormone Treatment

**DOI:** 10.1210/clinem/dgaa412

**Published:** 2020-06-27

**Authors:** Iris de Nie, Christel J M de Blok, Tim M van der Sluis, Ellis Barbé, Garry L S Pigot, Chantal M Wiepjes, Nienke M Nota, Norah M van Mello, Noelle E Valkenburg, Judith Huirne, Louis J G Gooren, R Jeroen A van Moorselaar, Koen M A Dreijerink, Martin den Heijer

**Affiliations:** 1 Department of Endocrinology, Amsterdam UMC, VU University Medical Centre, Amsterdam, the Netherlands; 2 Centre of Expertise on Gender Dysphoria, Amsterdam UMC, VU University Medical Centre, Amsterdam, the Netherlands; 3 Department of Urology, Amsterdam UMC, VU University Medical Centre, Amsterdam, the Netherlands; 4 Department of Pathology, Amsterdam UMC, VU University Medical Centre, Amsterdam, the Netherlands; 5 Department of Obstetrics and Gynecology, Amsterdam UMC, VU University Medical Centre, Amsterdam, the Netherlands

**Keywords:** prostate cancer, transgender, androgen deprivation, gender dysphoria

## Abstract

**Context:**

Trans women (male sex assigned at birth, female gender identity) mostly use antiandrogens combined with estrogens and can subsequently undergo vaginoplasty including orchiectomy. Because the prostate remains in situ after this procedure, trans women are still at risk for prostate cancer.

**Objective:**

To assess the incidence of prostate cancer in trans women using hormone treatment.

**Design:**

In this nationwide retrospective cohort study, data of participants were linked to the Dutch national pathology database and to Statistics Netherlands to obtain data on prostate cancer diagnosis and mortality.

**Setting:**

Gender identity clinic.

**Participants:**

Trans women who visited our clinic between 1972 and 2016 and received hormone treatment were included.

**Main Outcome Measures:**

Standardized incidence ratios (SIRs) were calculated using the number of observed prostate cancer cases in our cohort and the number of expected cases based on age-specific incidence numbers from the Netherlands Comprehensive Cancer Organization.

**Results:**

The study population consisted of 2281 trans women with a median follow-up time of 14 years (interquartile range 7-24), and a total follow-up time of 37 117 years. Six prostate cancer cases were identified after a median 17 years of hormone treatment. This resulted in a lower prostate cancer risk in trans women than in Dutch reference males (SIR 0.20, 95% confidence interval 0.08-0.42).

**Conclusions:**

Trans women receiving androgen deprivation therapy and estrogens have a substantially lower risk for prostate cancer than the general male population. Our results support the hypothesis that androgen deprivation has a preventive effect on the initiation and development of prostate cancer.

Transgender people experience an incongruence between the sex assigned at birth and their experienced or expressed gender (1). People assigned male at birth who identify as male are defined as cis men, and those who identify as female are defined as trans women. In the Netherlands, the prevalence of gender dysphoria in birth-assigned males is approximately 1 in 2800 (2). Transgender people may choose medical treatment to align their physical characteristics with their experienced gender, including gender-affirming hormone treatment and gender-affirming surgery. Hormone treatment for trans women consists of antiandrogens combined with estrogens (3). Although gender-affirming hormone treatment is generally considered safe, there may be risks and side effects, such as thromboembolic events or the development of sex hormone-related cancers (eg, breast cancer) 4-6).

Gender-affirming surgery involves bilateral orchiectomy, often combined with vaginoplasty and sometimes with breast augmentation (7). Although the prostate is biologically a male organ, prostatectomy is not performed during gender-affirming surgery because of the potential significant complications, such as incontinence. Therefore, trans women remain at risk for prostatic diseases after this procedure. It has been assumed that sex hormones, and androgens in particular, are involved in the pathogenesis of prostate cancer, because of the physiological dependency of prostate cells on androgens for functioning and proliferation (8, 9). In metastasized or advanced prostate cancer, androgen deprivation therapy is used to slow the progression of the disease (10). However, a large meta-analysis showed no association between endogenous serum testosterone levels and prostate cancer incidence nor did it show an increased prostate cancer risk in hypogonadal men using testosterone replacement therapy (11).

Androgen deprivation therapy in cis men is primarily used in patients diagnosed with advanced prostate cancer and only sporadically for other indications, such as to control sex impulses in patients with severe paraphilias (12). Therefore, there are currently very limited data available about a potential preventive effect of long-term androgen deprivation on the occurrence of prostate cancer.

The primary aim of this study was to investigate the incidence of prostate cancer in trans women receiving androgen deprivation therapy and estrogens. Furthermore, this study gives a unique opportunity to study the potential preventive effect of androgen deprivation on the initiation and development of prostate cancer in general.

## Materials and Methods

### Study design and data collection

For this retrospective cohort study, data on subjects were obtained from their medical files including medical history, age at the start of hormone treatment, documented hormone use, and data on gender-affirming surgery. This database was linked to the Nationwide Network and Registry of Histopathology and Cytopathology in the Netherlands (PALGA) to obtain data regarding prostate cancer histology and the date of prostate cancer diagnosis (13). Data with regard to mortality were obtained from Statistics Netherlands to calculate follow-up time (14).

The study protocol was assessed by the Ethics Review Board of the VU University Medical Centre Amsterdam. It was concluded that the Medical Research Involving Human Subjects Act did not apply to this study, and necessity for informed consent was waived because of the retrospective design and the large study population. Transgender people or the public were not involved in the design, or conduct, or reporting, or dissemination plans of our research.

### Study population

All individuals who visited the gender identity clinic of the Amsterdam UMC between 1972 and 2016 were identified. This cohort has been previously described as the Amsterdam Cohort of Gender Dysphoria (2). For this study, only trans women who received hormone treatment were included. People who never used hormone treatment, or the start dates of hormone treatment were unknown, who were under 18 years of age at the time of the study, or who used female and male hormones alternatingly were excluded. Since data on prostate cancer diagnosis were obtained from PALGA, which covers histopathologic diagnoses since 1991, people were also excluded when their last visit to our clinic was before 1991 (13).

The prescribed hormone treatment for trans women generally consisted of a combination of antiandrogens and estrogens. In our cohort, the most commonly prescribed medication to achieve androgen deprivation was cyproterone acetate, only sporadically spironolactone was used. People were advised to discontinue antiandrogenic treatment after bilateral orchiectomy. Types of prescribed estrogens included estradiol valerate, estradiol patches, estradiol gel, ethinyl estradiol, conjugated estrogens, estradiol implants, and estradiol injections. From 2001 onward, mainly estradiol valerate, estradiol patches, or estradiol gel were used. People who were younger than 18 years when they started hormone treatment had often only used a gonadotropin-releasing hormone agonist, namely triptorelin, prior to the start with estrogens.

### Statistical analysis

Characteristics of the cohort are expressed as means with standard deviation when normally distributed, and as medians with interquartile range (IQR) when non-normally distributed. When the cohort consists of less than 10 individuals, ranges are given instead of IQRs. To calculate the incidence rate for prostate cancer in our cohort, follow-up time was defined as years from the start date of hormone treatment until either prostate cancer diagnosis, date of death, or the end of the study period (January 25, 2019). To calculate age-adjusted standardized incidence ratios (SIRs), we used the observed cases of prostate cancer and the expected cases based on age-specific incidence rates obtained from the Netherlands Comprehensive Cancer Organization (15). Since the Netherlands Comprehensive Cancer Organization generates prostate cancer incidence rates using data from the same source (PALGA), this allows for a reliable comparison. SIRs with 95% confidence interval (95% CI) were calculated using a mid-exact *P* test. Subgroup analyses were performed for trans women who underwent orchiectomy, and for those who did not. For these analyses, follow-up time of people who underwent orchiectomy was calculated from the date of surgery until one of the previously mentioned terminating events. Lastly, SIRs (95% CI) were calculated for different age categories.

STATA Statistical Software, version 14.1 (Statacorp, College Station, TX, USA) and OpenEpi version 3.01 (www.OpenEpi.com) were used for statistical analyses.

## Results

**Figure 1.**
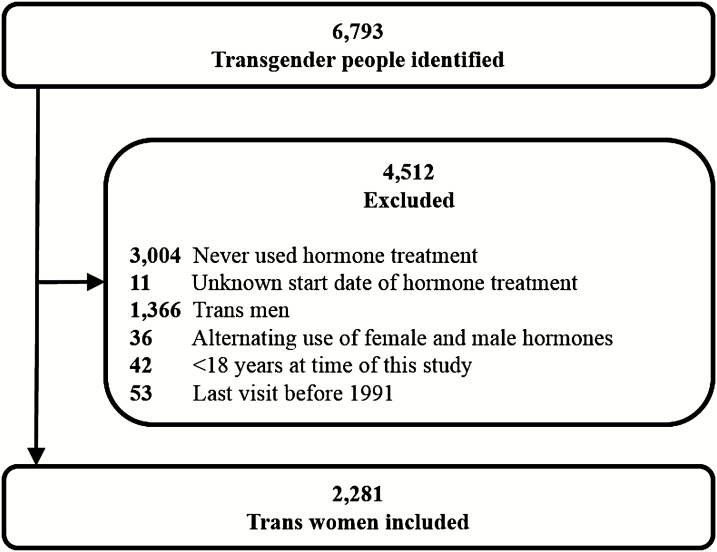
Study flowchart.

A total of 6793 individuals were identified, of whom 4432 were birth-assigned males and 2361 were birth-assigned females. After applying the inclusion and exclusion criteria, 2281 trans women were included in this study (Fig. 1). The median age at start of hormone treatment was 31 years (IQR 23-41). The median follow-up time was 14 years (IQR 7-24) per person and the total follow-up time of the entire cohort was 37 117 years. Table 1 shows the characteristics of the entire study cohort.

**Table 1.** Characteristics of study cohort

	Total cohort (n = 2281)
Age at time of study (years)	50 (37–59)
Age at start of hormonal treatment (years)	31 (23–41)
Body mass index (kg/m^2^)	22.7 (20.5–25.6)^*a*^
% Caucasian ethnicity	96.7 (n = 1579)^*b*^
% (former) smokers	39.0 (n = 890)
% orchiectomy	68.9 (n = 1572)
Follow-up time (years)	14 (7–24)
Total follow-up time (years)	37 117

Values are medians (interquartile ranges) unless stated otherwise. Data available for ^*a*^1357 and ^*b*^1633 people of cohort.

Six people were diagnosed with prostate cancer, after a median 17 years (range 10-24) of hormone treatment. The 6 trans women with prostate cancer had started hormone treatment at a median age of 47 years (range 38-58). Four of these 6 individuals had undergone orchiectomy, median 11 years (range 2-14), prior to the prostate cancer diagnosis. The median age at time of diagnosis was 64 years (range 53-77).

Histology reports of the 6 prostate cancer cases in our cohort showed adenocarcinoma in all cases. Gleason scores, the recommended prostate cancer grading system, were available for 5 trans women (16). In all cases, there was at least 1 biopsy with a Gleason score of 7 or higher, suggesting a tumor with intermediate risk or higher. With a median of 18 ng/mL (range 5-1722), serum levels of prostate-specific antigen (PSA) were elevated at time of diagnosis in all cases.

Based on age-specific incidence rates, the number of expected prostate cancer cases in our cohort was 30. Since only 6 cases were observed in our cohort (16.2 cases per 100 000 years), the prostate cancer risk was considerably lower than in Dutch cis males (SIR 0.20, 95% CI 0.08-0.42). This preventive effect holds in a subgroup analysis of trans women who underwent orchiectomy as part of their gender-affirming treatment (SIR 0.17, 95% CI 0.05-0.40, Table 2).

**Table 2.** Standardized incidence ratios for prostate cancer in trans women using hormone treatment

	Follow-up time (years)	Observed cases	Dutch incidence rates (per 100 000 persons, per year) (15)	Expected cases	Standardized incidence ratio (95% confidence interval)
Age categories					
<30 years	7,190	0	0	0	—
30-44 years	14 800	0	0.32	0	—
45-59 years	13 429	1	58.56	7	0.14 (0.01-0.70)
60-74 years	4,428	4	490.54	20	0.20 (0.06-0.48)
>75 years	535	1	567.68	3	0.33 (0.02-1.64)
Overall (n = 2,281)	37 117	6	—	30	0.20 (0.08-0.42)
Subgroup analyses					
Hormone treatment with orchiectomy (n = 1572)	26 048	4	—	24	0.17 (0.05-0.40)
Hormone treatment without orchiectomy (n = 709)	6796	2	—	5	0.44 (0.07-1.47)

We were unable to perform analyses on different hormone treatment protocols, since, on the one hand, many trans women change often between different types of prescribed estrogens over time and, on the other hand, they mostly use cyproterone acetate as antiandrogenic treatment.

## Discussion

Our study shows a 5-fold decrease in prostate cancer risk in trans women using hormone treatment compared with the general male population of similar age. This observation provides new insight in the relationship between testosterone and prostate cancer risk. Where previously no association was found between serum testosterone concentrations and the incidence of prostate cancer, our results show that very low serum testosterone concentrations have a substantial preventive effect on the initiation and development of prostate cancer.

In this study, we linked the cohort of trans women with nationwide registries on prostate cancer and mortality. Therefore, we feel that the incidence we found of 16.2 prostate cancer cases per 100 000 years is a reliable estimate. In 2014, Gooren and Morgentaler reported an incidence of 2.0 prostate cancer cases per 100 000 person years in trans women (17). This is lower than the reported incidence in our current study and is likely due to the absence of information on prostate cancer cases diagnosed in other centers and the lack of mortality data leading to an overestimation of follow-up time. Another cohort study showed a much higher incidence of 72 prostate cancer cases per 100 000 person years for trans women (18). However, the higher incidence of prostate cancer in this American cohort may be explained by the fact that 38% of their study population consisted of trans women who had not undergone gender-affirming hormone treatment and were, therefore, not androgen deprived (19).

Our study is not only relevant for health management of trans women. It also provides a unique insight into the relationship between serum testosterone levels and the occurrence of prostate cancer. A paradox in the current knowledge is that, on the one hand, androgen deprivation slows the progression of metastasized or advanced prostate cancer, while, on the other hand, higher endogenous serum testosterone or elevating testosterone concentrations in hypogonadal men do not increase prostate cancer risk (10, 11). A proposed theory suggests that the relationship between androgens and prostate cancer follows a saturation curve, as applies to the relation between PSA and testosterone levels (20). This model states that if all androgen receptors are bound, a further increase in serum androgen concentrations produces no additional biological effects. Below this point of saturation, androgen concentration serves as the rate-limiting step in prostate tissue proliferation (20). The saturation model accounts for the slowed progression of prostate cancer by suppression of androgens below castration levels. As total testosterone concentrations in trans women receiving hormone treatment are generally below 1.7 nmol/L during hormone treatment, the observed low incidence implies that the saturation model might also apply to the initiation and development of prostate cancer (3).

We also have to take the role of estrogen treatment that is used by these individuals into account. One might argue that the incidence of prostate cancer is not only influenced by androgen deprivation but also by the use of estrogens. Estrogens contribute to androgen deprivation through suppression of the hypothalamic–pituitary–gonadal axis and were initially used in prostate cancer treatment before the implementation of luteinizing hormone-releasing hormone agonists (10). However, some theories also suggest a possible stimulating role of estrogens in the pathogenesis of prostate cancer (21, 22). Estrogen receptors are expressed in the human prostate. With respect to cell-autonomous actions of estrogens in prostate tissue, both pro- and antiproliferative effects have been reported in the literature (23). Estrogen receptor alpha has been shown to stimulate prostate cancer growth in preclinical models. Conversely, loss of expression of estrogen receptor beta has been reported in prostate cancer tissues, implying a role as a tumor suppressor (22). The exact role of estrogens in the pathogenesis of prostate cancer and its effect on the occurrence of the disease remains unclear and might be an interesting topic for future research.

In our cohort, 6 trans women developed prostate cancer while receiving hormone treatment. It may have been possible that these 6 individuals harbored small foci of subclinical prostate cancer prior to the start of hormone treatment. Autopsy studies in healthy subjects who died of trauma have shown microscopic foci of prostate cancer and high-grade prostatic intraepithelial neoplasia from the third decade of life onwards (24-26). The lesions become more frequent and extensive as age increases. This complements our observation that trans women with prostate cancer had a median age of 47 years at start of hormone treatment. Trans women in previously reported cases of prostate cancer also started hormone treatment at an older age (27). Possibly androgen deprivation slowed further progression of these carcinogenic foci for many years. The mechanisms of how these foci eventually might have evolved into prostate cancer, despite androgen deprivation, are perhaps similar to proposed mechanisms of the transition from hormone-sensitive to castration-resistant prostate cancer. Different theories suggest that castration-resistant prostate cancers have developed mechanisms that enable them to use steroids from the circulation more efficiently through de novo androgen synthesis, changed function of the androgen receptor, or even through estrogen receptor signaling pathways (21, 22, 28).

European guidelines advise against systematic population-based PSA screening for prostate cancer, since it does not increase survival and causes overtreatment (29). Following these guidelines, routine population-based PSA screening, in both cis men and trans women, is not performed in the Netherlands. PSA testing is only recommended in people with an elevated risk of prostate cancer after counselling on the potential risks and benefits (30). Given the low incidence of prostate cancer and lack of PSA reference values in this population, there is even less reason to perform routine screening in trans women. However, it remains important that trans women and their healthcare providers are aware of the presence of the prostate and the possibility of developing prostate cancer despite low serum androgen levels.

This study provides novel insights into prostate cancer risk in trans women receiving androgen deprivation and estrogen treatment. The major strengths of our study include the large cohort size consisting of people with a wide age range, and the validation of our data by linking our cohort to the Dutch national pathology database. Also, our study has a long follow-up period that was adequately calculated using data on mortality from Statistics Netherlands. Furthermore, since the current practice for prostate cancer screening in the Netherlands is the same for cis men and trans women, that is, no routine PSA testing, the role of detection bias in this study seems to be very limited.

A limitation of this study is the lack of information about the clinical symptoms of the 6 trans women with prostate cancer that led to the diagnosis, since the majority were diagnosed and treated in other hospitals than our clinic. Furthermore, information about family history, hormone use, and lifestyle was missing or incomplete due to the retrospective design of our study. In particular, data on family history would have provided more insight into the genetic susceptibility for prostate cancer, which is known to be an important risk factor (31). Although the influence of these risk factors on prostate cancer should not be underestimated, the most profound difference between our cohort and the reference population is gender-affirming treatment with antiandrogens and estrogens and eventually orchiectomy.

For future studies, it would be worthwhile to investigate the clinical symptoms in trans women leading to prostate cancer diagnosis. Further research is needed to understand the pathogenesis of prostate cancer in trans women receiving hormone treatment and how this influences treatment outcome compared with cis males.

## Conclusions

This large nationwide cohort study in trans women receiving hormone treatment showed a 5-fold decrease in prostate cancer risk compared with the general male population of similar age. This observation confirms our hypothesis that androgen deprivation has a preventive effect on the initiation and development of prostate cancer in general. Although the risk is much lower in trans women, prostate cancer in this population still occurs. Trans women, their general practitioners, and other healthcare providers should be aware of the possibility of the development of prostate cancer despite low serum androgen levels.

## Abbreviations

CI: confidence interval
IQR: interquartile range
PALGA: Nationwide Network and Registry of Histopathology and Cytopathology in the Netherlands
PSA: prostate-specific antigen
SIR: standardized incidence ratio

## Additional Information

***Disclosure Statement:*** The authors have nothing to disclose.

***Data Availability:*** Restrictions apply to the availability of data generated or analyzed during this study to preserve patient confidentiality or because they were used under license. The corresponding author will on request detail the restrictions and any conditions under which access to some data may be provided.
